# Ediacaran developmental biology

**DOI:** 10.1111/brv.12379

**Published:** 2017-11-03

**Authors:** Frances S. Dunn, Alexander G. Liu, Philip C. J. Donoghue

**Affiliations:** ^1^ School of Earth Sciences University of Bristol Life Sciences Building, 24 Tyndall Avenue, Bristol BS8 1TQ U.K.; ^2^ British Geological Survey Nicker Hill, Keyworth, Nottingham NG12 5GG U.K.

**Keywords:** Metazoa, development, evolution, Ediacaran, Bilateria, Eumetazoa

## Abstract

Rocks of the Ediacaran System (635–541 Ma) preserve fossil evidence of some of the earliest complex macroscopic organisms, many of which have been interpreted as animals. However, the unusual morphologies of some of these organisms have made it difficult to resolve their biological relationships to modern metazoan groups. Alternative competing phylogenetic interpretations have been proposed for Ediacaran taxa, including algae, fungi, lichens, rhizoid protists, and even an extinct higher‐order group (Vendobionta). If a metazoan affinity can be demonstrated for these organisms, as advocated by many researchers, they could prove informative in debates concerning the evolution of the metazoan body axis, the making and breaking of axial symmetries, and the appearance of a metameric body plan. Attempts to decipher members of the enigmatic Ediacaran macrobiota have largely involved study of morphology: comparative analysis of their developmental phases has received little attention. Here we present what is known of ontogeny across the three iconic Ediacaran taxa Charnia masoni, Dickinsonia costata and Pteridinium simplex, together with new ontogenetic data and insights. We use these data and interpretations to re‐evaluate the phylogenetic position of the broader Ediacaran morphogroups to which these taxa are considered to belong (rangeomorphs, dickinsoniomorphs and erniettomorphs). We conclude, based on the available evidence, that the affinities of the rangeomorphs and the dickinsoniomorphs lie within Metazoa.

## I. INTRODUCTION

Among multicellular eukaryotes, Metazoa are unique in exploring a broad range of diverse body plans. Assisted by their ability to undergo coordinated embryogenesis (Valentine, Tiffney, & Sepkoski, 1991), and free from the restrictions of rigid cell walls, animals have evolved well over 100 distinct cell types [compared to ∼7 in fungi and kelps and ∼30 in higher plants (Bonner, 1988)], and have arranged them into diverse tissue types, physiological systems, and morphological structures. Animals are therefore among the most biologically complex organisms. Elucidating the developmental processes that underpin this complexity is a major challenge for contemporary evolutionary and developmental biology.

Molecular clock estimates suggest that animals originated ∼700–800 million years ago (Ma) (dos Reis *et al*., 2015), but unequivocal fossil evidence for animals is not found until closer to ∼541 Ma (e.g. Erwin *et al*., 2011; Cunningham *et al*., 2017). Some of the most likely candidates for early animal fossils are found within the Ediacaran Biota; an enigmatic assemblage of largely soft‐bodied macroscopic organisms that spans the ∼40 million year interval immediately prior to the Cambrian Period (Fedonkin *et al*., 2007; Cunningham *et al*., 2017). Many of these organisms, which are typically preserved only as impressions of their external surfaces, are united by a body plan composed of self‐repeating morphological units. Their fossil remains possess few morphological characters that are diagnostic of any particular phylogenetic affinity, and multiple competing hypotheses for where they lie within Eukarya have been proposed since their initial description (summarised in Xiao & Laflamme, 2009; Budd & Jensen, 2017), including the suggestion that they represent an entirely distinct Kingdom Vendobionta (Seilacher, 1989, 1992). This latter hypothesis later softened to consider the Ediacaran Biota as an extinct phylum within total‐group Metazoa or total‐group Eumetazoa (Buss & Seilacher, 1994); a view not substantially different from the current broad consensus that these Ediacaran organisms are allied to early‐branching lineages of Metazoa or Eumetazoa (e.g. Xiao & Laflamme, 2009; Budd & Jensen, 2017). Surprisingly, in many cases this consensus does not rest on material evidence of metazoan affinity but, rather, on an implicit assumption that the organisms are total‐group metazoans. As a result, Ediacaran taxa are invoked in debate on the origin and evolution of metazoan developmental novelties, including the specification of primary body axes, the making and breaking of axial symmetries, and the appearance of metamerism and/or segmentation (e.g. Malakhov, 2004). Determining the correct phylogenetic position of Ediacaran macrofossil taxa, or even being able to provide convincing positive evidence for an unquestionably metazoan placement, is therefore a challenge with significant consequences for understanding the evolution of metazoan development and morphogenesis.

It is perhaps surprising that although developmental insights can be gleaned from Ediacaran fossil assemblages, Ediacaran developmental biology remains in its infancy. The little work that has been done, based on the premise that ontogenetic characters are considered to have been conserved across evolutionary time, demonstrates the potential power of morphogenesis in testing established hypotheses of affinity (e.g. Antcliffe & Brasier, 2007: Gold *et al*., 2015). Investigation of morphogenesis in Ediacaran taxa also has the potential to constrain hypotheses of developmental evolution associated with the evolutionary emergence of animals, and to test models of trait evolution that are currently based only on theoretical predictions. Here we review the existing data and interpretations regarding morphogenesis in key Ediacaran macro‐organisms, and use this information to constrain hypotheses of their evolutionary relationships to extant eukaryotic groups.

## II. THE SEMANTICS OF EDIACARAN MORPHOGENESIS

Describing ontogeny in fossil organisms can be problematic (e.g. Hone, Farke, & Wedel, 2016). Many extant organisms display some form of ontogenetic shift (Paris & Laudet, 2008) and this is often used to distinguish between juvenile and adult individuals. However, such shifts are difficult to identify with certainty in extinct organisms, and have typically not been recognised in Ediacaran fossil taxa, whose adult and juvenile stages have largely been distinguished based only on the size of the specimens (e.g. Liu *et al*., 2012). Moreover, many extant clades, including several metazoan groups to which members of the Ediacaran macrobiota have been compared, exhibit a morphologically distinct juvenile stage that bears little resemblance to the adult form (e.g. the planula larvae of Cnidaria). Discrimination of adults and juveniles among Ediacaran macrofossils is not, therefore, something that we can necessarily expect to achieve, and such terms should be avoided. The alternative use of ‘size classes’ is both arbitrary and potentially subject to change as new specimens are described. Allocation of specimens to ‘generations’ is another possibility (see Mitchell *et al*., 2015), but at least some bedding‐plane assemblages of Ediacaran macro‐organisms are considered to reflect only single generations, despite large variance in size (Darroch, Laflamme, & Clapham, 2013; although see Wilby, Kenchington, & Wilby, 2015). The simplest and most defensible strategy is to consider how the size of a fossil relates to smaller and larger specimens of the same species, and to make the reasonable assumption that larger individuals would have been older, or at least further developed, than smaller individuals (see Fedonkin, 2002; Narbonne, 2004; Flude & Narbonne, 2008).

Understanding the difference between pattern and process is also essential when considering growth in fossil taxa. It is clear that many Ediacaran taxa were composed of multiple units, which have at various points been termed branches, modules, units, isomers or segments. All of the taxa that we address have been considered to grow either by inflation (wherein a particular unit increases in size), ‘insertion’ (the sequential addition of units to an organism), or a combination of these (see Table 1 to compare the published distribution of these strategies across Ediacaran taxa). However, process terms must have a basis in ontological data (Jardine, 1969) and inferences of process should be evidenced and rationalised from assemblages of individuals representing different developmental stages. New structures and units can be added during the development of multicellular organisms in a variety of patterns, but this invariably occurs through differentiation of existing cells and tissues. Insertion of units, in the sense that it is described in Ediacaran macro‐organisms, does not occur in development, except in a metaphorical sense. Unfortunately, the metaphorical concept of unit insertion is at risk of being reified as a literal process in the interpretation of these fossils. Thus, we recommend use of the term ‘differentiation’ in place of ‘insertion’. This ensures that we do not limit comparisons to only those extant taxa that show *de novo* addition of new units. We use the term ‘insertion’ when summarising previous developmental studies of Ediacaran taxa in the following sections, but then revert to use of ‘differentiation’ from Section IV onwards.

**Table 1:** Summary of inflationary and ‘insertional’ (here renamed ‘differentiation’, see Section II for details) styles of growth across taxa belonging to the Ediacaran morphogroups Rangeomorpha, Dickinsoniomorpha and Erniettomorpha (sensu Erwin et al., 2011). Inflation is documented as minimal (if the organism is considered to grow almost exclusively by ‘insertion’), allometric (if units of the organism appear to inflate at different rates or to different degrees), isometric (if units of the organism appear to inflate at a constant rate to one another, maintaining overall shape), or simply present (if no further information on the degree of inflation is given). Differentiation (‘insertion’) is either noted as observed or not‐observed. Empty cells represent the absence of previously published data

Morphotype	Taxon	Inflation	Differentiation	References
Rangeomorph	*Charnia masoni*	Allometric	Observed	Brasier, Antcliffe, & Liu (2012) and Antcliffe & Brasier (2008)
Rangeomorph	*Vinlandia antecedens*			
Rangeomorph	*Trepassia wardae*	Minimal	Observed	Narbonne *et al*. (2009)
Rangeomorph	*Beothukis/Culmofrons plumosa*	Present	Not‐observed	Laflamme, Flude, & Narbonne (2012) and Liu, Matthews, & McIlroy (2016)
Rangeomorph	*Beothukis mistakensis*	Allometric	Not‐observed	Laflamme *et al*. (2012) and Liu *et al*. (2016)
Rangeomorph	*Avalofractus abaculus*			
Rangeomorph	*Fractofusus andersoni*	Isometric	Not‐observed	Darroch *et al*. (2013) and Gehling & Narbonne (2007)
Rangeomorph	*Fractofusus misrai*	Allometric/Isometric	Not‐observed	Darroch *et al*. (2013) and Gehling & Narbonne (2007)
Rangeomorph	*Bradgatia linfordensis*			
Rangeomorph	*Bradgatia sp*.	Present	Not‐observed	Flude & Narbonne (2008)
Rangeomorph	*Primocandelabrum hiemaloranurn*			
Rangeomorph	*Primocandelabrum sp*.			
Rangeomorph	*Hapsidophyllas flexibilis*			
Rangeomorph	*Frondophyllas grandis*			
Rangeomorph	*Plumeropriscum hofmanni*			
Rangeomorph	*Pectinifrons abyssalis*	Present	Observed	Bamforth, Narbonne, & Anderson (2008)
Dickinsoniomorph	*Andiva ivantsovi*	Isometric		Fedonkin (2002)
Dickinsoniomorph	*Dickinsonia costata*	Allometric	Observed	Hoekzema *et al*. (2017), Evans, Droser, & Gehling (2017), Gold *et al*. (2015), Ivantsov (2007), Retallack (2007), Runnegar (1982)
Dickinsoniomorph	*Dickinsonia lissa*	Present		Ivantsov (2007)
Dickinsoniomorph	*Dickinsonia rex*	Present	Observed	Ivantsov (2007) and Retallack (2007)
Dickinsoniomorph	*Dickinsonia tenuis*	Present	Observed	Ivantsov (2007) and Retallack (2007)
Dickinsoniomorph	*Windermeria aitkeni*			
Dickinsoniomorph	*Yorgia waggoneri*		Observed	Ivantsov (2007)
Erniettomorph	*Ernietta plateauensis*	Present	Not observed	Ivantsov *et al*. (2016) and Dzik (1999)
Erniettomorph	*Nasepia altae*			
Erniettomorph	*Palaeoplatoda segmentata*			
Erniettomorph	*Phyllozoon hanseni*			
Erniettomorph	*Pteridinium simplex*	Present	Observed	Grazhdankin & Seilacher (2002)
Erniettomorph	*Swartpuntia germsi*			
Erniettomorph	*Valdainia plumosa*			

Finally, we note that previous rangeomorph taxonomic schemes have focused on assumed polarity of growth, considering various organisms as either unipolar, bipolar or multipolar (Brasier *et al*., 2012). However, the assumption that growth is occurring in the positions ascribed by these terms remains untested (in an ontogenetic sense) in many rangeomorphs. We prefer here to use morphologically descriptive terminology (as opposed to morphogenetically descriptive). Previous attempts at morphological description have considered fronds to be constructed of petalodia and petaloids (Laflamme & Narbonne, 2008), but such terminology has more recently been considered inappropriate, since its correct deployment is also somewhat reliant on a complete understanding of an organism's life history (Brasier *et al*., 2012). We therefore introduce the terms ‘uniterminal’, ‘biterminal’ and ‘multiterminal’ as morphological descriptors of the number of distal tips the frond possesses (not including the stem or holdfast). In practical application, previous groupings of rangeomorphs remain the same, but the new terms here are based entirely on morphological features, and avoid all assumptions regarding morphogenesis.

## III. ONTOGENY IN EDIACARAN MORPHOGROUPS

To date, ∼200 Ediacaran macrofossil taxa have been described (Fedonkin *et al*., 2007), and multiple attempts have been made to group these within sub‐groups of closely related organisms. Initially, many Ediacaran taxa were considered members of extant animal clades (e.g. Glaessner, 1984), but more recently they have instead been grouped according to morphological similarity (Erwin *et al*., 2011; Grazhdankin, 2014), with such groupings representing grades (rather than clades) of organism. We focus our study on fossils considered to belong to three widely recognised morphogroups that together include many of the most contentious members of the Ediacaran biota: the rangeomorphs, dickinsoniomorphs and erniettomorphs. Members of these groups have all, at some point, been interpreted as animals, with some researchers considering members of all three groups to share a self‐similar body plan, perhaps indicating a common evolutionary history (Seilacher, 1989, 1992; Buss & Seilacher, 1994). We favour the use of morphogroups because it confers phylogenetic neutrality, but we note the possibility that unrelated taxa may be grouped together within such morphogroups, potentially obscuring phylogenetic signal. These concerns may be allayed by independent attempts to resolve the phylogenetic relationships among the Ediacaran grades that have provided some support for the biological reality of some morphogroups (Dececchi *et al*., 2017). Regardless, while we acknowledge that the composition of these morphogroups may not be entirely coherent in phylogenetic terms, we consider them to provide a useful framework within which to sample the disparity of Ediacaran macro‐organism body plans.

Hoyal Cuthill & Conway Morris (2017) have attempted to explain variation among Ediacaran frondose organisms as a consequence of ecophenotypism, produced in response to variation in nutrient levels in the water column across different palaeoenvironments. This suggestion potentially introduces an alternative explanation for morphological variation otherwise interpreted as taxonomic or ontogenetic. While we recognise the presence of some ecophenotypic variation within Ediacaran assemblages, we note that population‐level studies of frondose organisms continue to document discrete taxonomic variation (e.g. Kenchington & Wilby, 2017). Hoyal Cuthill & Conway Morris (2017) based their study on only three, anatomically discrete, specimens, representing taxa that are known to co‐occur on bedding planes (Narbonne *et al*., 2009), consistent with morphological variation existing within assemblages subject to similar palaeoenvironmental regimes. Until relationships between morphology and ambient nutrient levels can be demonstrated we consider size variation within Ediacaran taxa to reflect ontogeny.

### (1) Rangeomorpha

**Figure 1:**
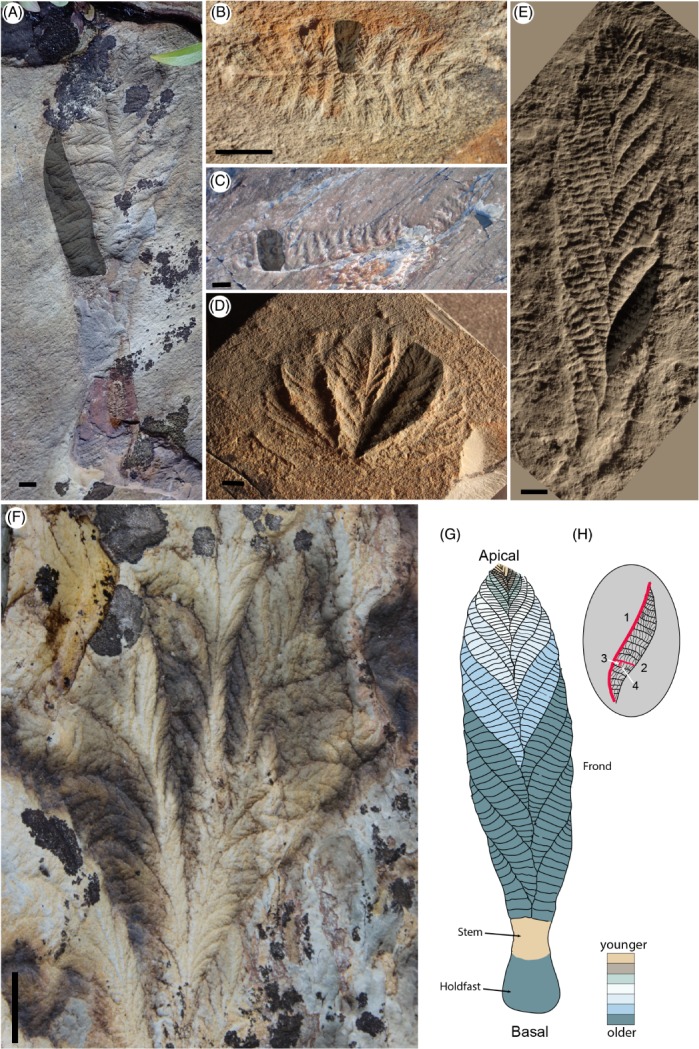
Ediacaran rangeomorph taxa. (A) Beothukis plumosa, Newfoundland, Canada. (B) Fractofusus andersoni, Newfoundland, Canada. (C) Pectinifrons abyssalis, Newfoundland, Canada. (D) Bradgatia sp., Newfoundland, Canada. (E) Charnia masoni, UK. (F) Higher‐order branching in an exceptionally preserved Bradgatia sp. specimen from Newfoundland. (G) Stylised interpretation of growth of primary branches in Charnia masoni. (H) The different orders of rangeomorph branches, and their arrangement within Charnia masoni: 1 = primary branch, 2 = secondary branch, 3 = tertiary branch and 4 = quaternary branch. Grey overlay in A–E indicates a primary branch. Scale bars: A, B, D and E = 10 mm, C = 5 cm.

Rangeomorpha (Fig. 1) encompasses organisms that share a body plan comprising one or multiple fronds constructed of serially repeated, leaf‐like, self‐repeating branches [see supplementary online material (SOM) of Erwin *et al*., 2011]. Rangeomorphs were seemingly sessile organisms that lived in deep‐ and shallow‐marine depositional environments, and are a stratigraphically long‐ranging morphogroup, spanning the interval ∼570–541 Ma (Boag, Darroch, & Laflamme, 2016; Pu *et al*., 2016). Rangeomorphs can be uniterminal (with one apparent distal terminus: e.g. *Charnia masoni*), biterminal (e.g. *Fractofusus*) or multiterminal (e.g. *Bradgatia*), and the arrangement of their branches has been proposed as a basis for distinguishing between taxa (Narbonne *et al*., 2009; Brasier *et al*., 2012). Morphogenesis has been considered most widely in the cosmopolitan taxon *Charnia masoni*, which possesses many features characteristic of rangeomorphs (Brasier & Antcliffe, 2004, 2009).

#### (a) Charnia masoni

*Charnia masoni* (Fig. 1E) is a uniterminal rangeomorph with a global late‐Ediacaran distribution, found in the UK (e.g. Wilby *et al*., 2015), Newfoundland (e.g. Laflamme *et al*., 2007), northwestern Canada (Narbonne *et al*., 2014), South Australia (e.g. Gehling & Droser, 2013), the White Sea of Russia (Fedonkin, 1990), and Siberia (e.g. Grazhdankin *et al*., 2008). It has been variously compared to algae (Ford, 1958), fungi (Peterson, Waggoner, & Hagadorn, 2003), stem‐metazoans (Budd & Jensen, 2017), pennatulacean cnidarians (Glaessner, 1984), or placed in a hypothetical non‐metazoan higher order group (Seilacher, 1989, 1992). Known *Charnia masoni* specimens range from ∼1 cm (Liu *et al*., 2012) to >65 cm (Boynton & Ford, 1995) in length, with size variants typically interpreted as different ontogenetic stages in the *Charnia* life cycle (e.g. Liu *et al*., 2012).

**Figure 2:**
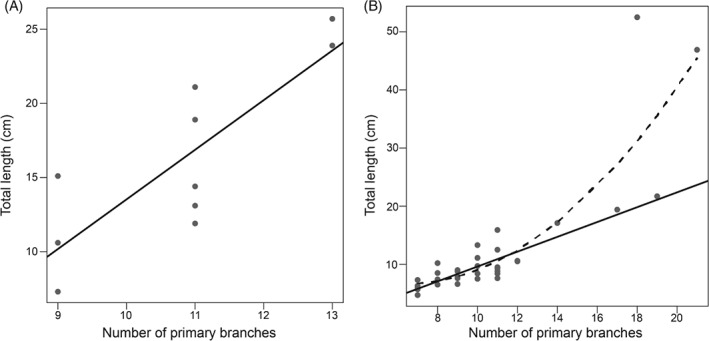
The length of Charnia masoni specimens plotted against the number of primary branches in specimens from: (A) Sword Point, Newfoundland, Canada (data from Laflamme et al., 2007) (data have been retrodeformed); (B) North Quarry Bed B, Charnwood Forest, Leicestershire, UK (data from Wilby et al., 2015) (data were not retrodeformed). Linear models represented by solid line (fitted through a subset of data in B – excluding the two largest specimens); broken line represents a second‐order polynomial model. Both populations show a linear relationship between specimen size and the number of primary branches up to specimens 49 cm in length [P = 0.003429 and P = 5.327 × 10^−11^ for the Laflamme et al. (2007) and Wilby et al. (2015) data sets, respectively]; specimens larger than this are not explained by a linear model [the complete Wilby et al. (2015) data set is best fitted by a second‐order polynomial model, P = 1.579 × 10^−10^].

*Charnia* individuals of all sizes share a similar gross morphology, possessing multiple primary branches lying at a high angle along a glide plane of symmetry running through the central axis of the frond. The smallest frondose specimens appear to lack a stem, but all are considered to possess a sediment‐bound holdfast to anchor them to the seafloor (see fig. 4b in Liu *et al*., 2012). Primary branches in the smallest specimens range from five in a specimen of 1.0 cm length to seven in a specimen of 1.3 cm (Liu *et al*., 2012). Specimens longer than ∼7 cm possess a clear but short stem, which can exhibit branching down its length (fig. 2b in Laflamme *et al*., 2007; fig. 5.5 in Wilby *et al*., 2015), thus distinguishing this feature from the discrete ‘naked’ stem (i.e. lacking branched subdivisions) of other rangeomorphs (Laflamme *et al*., 2012) and non‐rangeomorph frondose taxa (e.g. *Charniodiscus*; Laflamme, Narbonne, & Anderson, 2004). There is a broad linear relationship between the number of primary branches in *Charnia masoni* and the overall length of the organism (Fig. 2), excepting the very largest specimens, which possess proportionally fewer branches than might be expected (Wilby *et al*., 2015). Primary branches increase in size as the organism increases in length (Wilby *et al*., 2015). No specimens of *Charnia* have been observed to possess greater than four hierarchical orders of branching. Previously collected ontogenetic data are derived only from primary branches and so development in higher branching orders, and the number of branch orders in the smallest specimens, has yet to be discerned.

These previous observations have led to interpretation of *Charnia* as growing by the ‘insertion’ and subsequent inflation of branches (Wilby *et al*., 2015). The consistent smaller size of primary branches at the apical region of individual fronds has been interpreted as evidence for a distal (apical) generative zone (Antcliffe & Brasier, 2007), with proximal primary branches (close to the holdfast) considered to have undergone a relatively longer inflation‐driven period of growth (fig. 2 in Antcliffe & Brasier, 2007). The proportionally lower number of primary branches in the largest specimens could represent an ontogenetic shift from an initial ‘insertion’‐driven stage of growth to a second inflation‐dominant interval with reduced rates of branch addition (Wilby *et al*., 2015). The largest *Charnia* specimens have been proposed as evidence for indeterminate growth, and seem to show no upper size constraints (Wilby *et al*., 2015).

**Figure 3:**
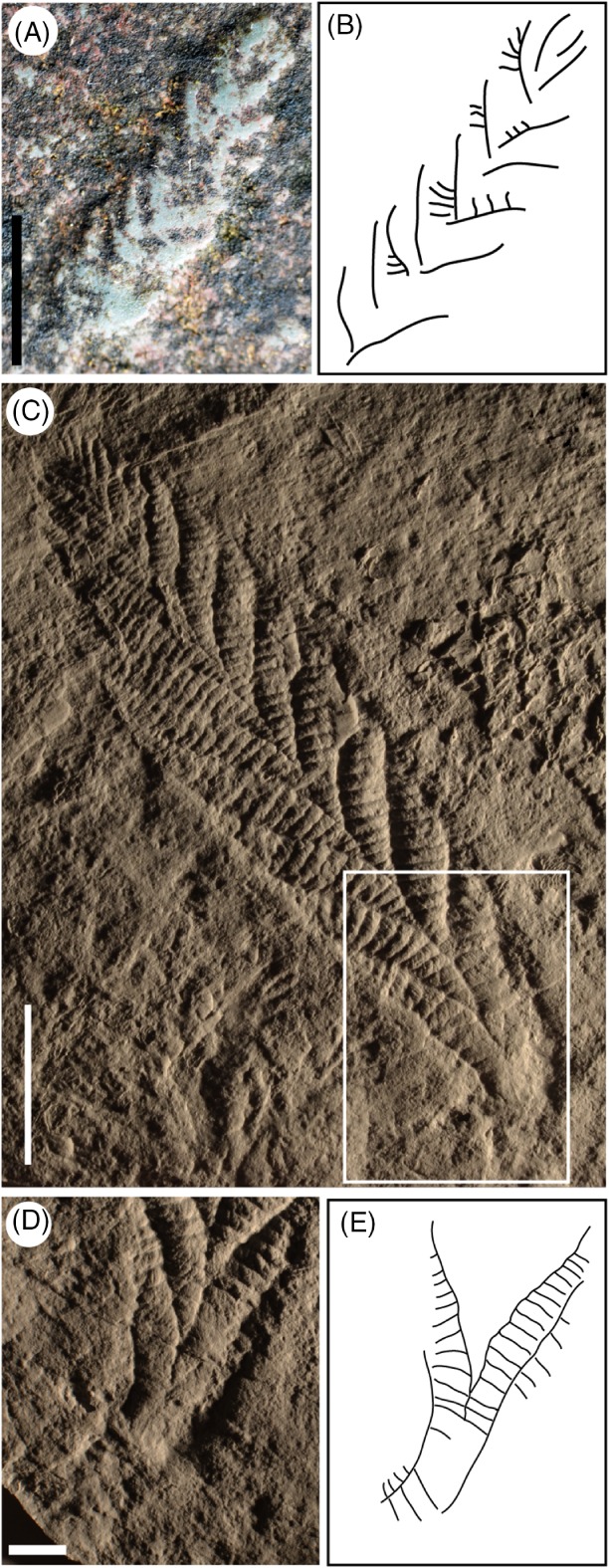
The development of the ‘stem’ region in Charnia masoni. (A, B) Charnia masoni from Pigeon Cove, Mistaken Point Ecological Reserve, Newfoundland, Canada (A) and outline of specimen (B). (C) Charnia masoni from Charnwood Forest, Leicestershire, UK. (D, E) Stem area (enlargement of boxed region in C) (D), and in outline (E). Illustrations to second‐order branch sub‐division. Scale bars: A = 5 mm, C = 5 cm, D = 10 mm.

The apparent absence of a stem in *Charnia* specimens less than ∼7 cm in length may indicate that a stem was not present in the youngest organisms (Fig. 3A, B). It is possible that the stem and holdfast were buried in small specimens, lying beneath the plane of preservation. However, these smallest specimens exhibit a ‘V’‐shaped termination at their base, with no suggestion of any downwards extension of the basal branches (Fig. 3A, B). If the stem was truly absent in early ontogenetic stages, emerging only later in the life cycle (Fig. 3C–E), the notion of *Charnia* possessing a single, distal growth tip (*sensu* Antcliffe & Brasier, 2007) becomes questionable since growth would also have occurred in a generative zone at the proximal end of the organism (depicted in Fig. 1G). Although *Charnia* undoubtedly possessed its smallest primary branches in the distal region of the frond (Antcliffe & Brasier, 2007), this observation alone is not proof of a solitary, distal, growth tip (see also Hoekzema *et al*., 2017).

#### (b) Ontogenetic trends across the rangeomorphs

Interpretations of growth across different rangeomorph taxa largely assume that branches underwent subdivision from a distal growth zone (Brasier & Antcliffe, 2009; Hoyal Cuthill & Conway Morris, 2014) (Table 1), and compare growth strategies across the rangeomorphs by considering inflationary growth and the appearance of new branches. In many uniterminal forms, growth appears to have proceeded in a similar way to that inferred in *Charnia* (e.g. *Trepassia wardae*; Laflamme *et al*., 2007), but with some variation in the total number of primary branches, for example the imposition of an upper limit to the number of primary branches in certain taxa (Laflamme *et al*., 2012; Liu *et al*., 2016).

In contrast to *Charnia*, *Fractofusus* (Fig. 1B) does not exhibit a clear linear relationship between the size of the organism and the number (and length) of primary branches (Gehling & Narbonne, 2007). In both described species of *Fractofusus*, primary branch bundles decrease in size distally in both directions along the growth axis, implying the presence of two distal growth tips (i.e. a bipolar growth axis) if it is assumed that the smallest branches are also the youngest (Seilacher, 1989; Brasier *et al*., 2012). *Fractofusus misrai* exhibits additional variance, with asymmetric ‘subsidiary’ branches emerging from between primary branches (Gehling & Narbonne, 2007).

*Bradgatia* sp. (Fig. 1D, F) from Newfoundland, Canada, is the best‐studied multiterminal rangeomorph, with four known morphotypes, each considered to represent a different ontogenetic stage (fig. 3.4 in Flude & Narbonne, 2008). Primary branch lengths vary within populations from ∼2 to 14 cm (fig. 8c in Flude & Narbonne, 2008), but do not appear to be tightly correlated with the morphotype‐based ontogenetic sequence proposed for the taxon (Flude & Narbonne, 2008). More branches are visible in larger, and therefore, presumably, older morphotypes of *Bradgatia* (the average number increasing from four to seven across the morphs; table 1 in Flude & Narbonne, 2008). However, it may be that the more diffuse form of the larger morphotypes means that more branches are visible, rather than that new branches were ‘inserted’ later in life (Flude & Narbonne, 2008). Within a single primary branch, the number of secondary branches does not increase with branch length, varying between 5 and 10 in most cases (Flude & Narbonne, 2008). Two hypotheses attempt to explain how the different orders of rangeomorph branches may have grown: (*i*) fractal growth, whereby one branch order reaches a critical size, triggering the development of the next, lower, order; and (*ii*) a true inflationary model, where all branch orders are always present and grow in concert (Flude & Narbonne, 2008). *Bradgatia* is the only rangeomorph interpreted to possess secondary growth tips, added non‐deterministically at the apex of large primary branches (Brasier & Antcliffe, 2009).

In summary, rangeomorphs have been considered to grow by one of two growth models: (*i*) the ‘insertion’ of new units and their subsequent inflation; or (*ii*) the inflation of new units without additional ‘insertion’ (Table 1; Gehling & Narbonne, 2007; Bamforth *et al*., 2008; Flude & Narbonne, 2008). *Charnia*, *Fractofusus* and *Bradgatia* all exhibit smaller primary branches in smaller specimens, and *Charnia* shows an increase in the number of primary branches over time (although such a relationship is not seen in known ontogenetic stages of all rangeomorph taxa). All rangeomorphs for which ontogeny has been considered are interpreted to have grown *via* emergence of branches either from distally located generative zones positioned at the ends of a single, central proximodistal axis (as seen in the uniterminal and biterminal rangeomorphs), or through a central axis and the production of lateral, secondary growth tips (i.e. *Bradgatia*). Although the different ontogenetic patterns described in rangeomorphs can show divergence from the pattern seen in *Charnia*, we find no developmental evidence that would preclude their inclusion within a single clade.

### (2) Dickinsoniomorpha

**Figure 4:**
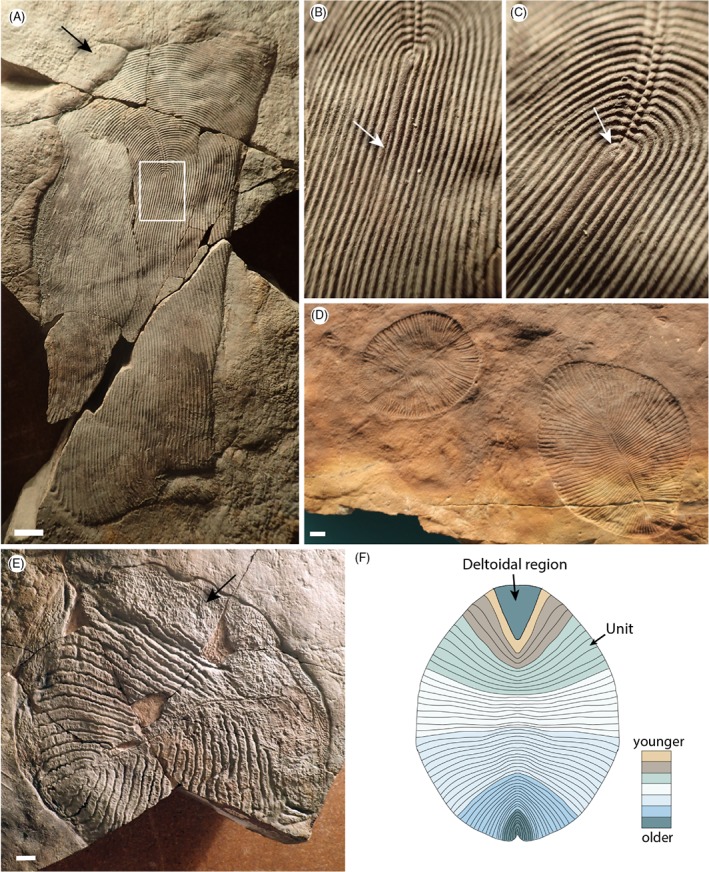
Ediacaran dickinsoniomorph taxa. (A) Andiva ivantsovi, White Sea, Russia. [Palaeontological Institute Moscow (PIN) specimen number 3993–5623]. (B, C) Enlargements of the boxed area in A. The areas of unit differentiation are indicated by white arrows, and undivided regions on Andiva and Yorgia are indicated by black arrows. (D) Dickinsonia costata, South Australia [South Australia Museum (SAM) specimen numbers P49354 and P49355]. (E) Yorgia waggoneri, White Sea, Russia (Holotype PIN 3993–5024). (F) Stylised interpretation of growth of Dickinsonia costata, following the growth model proposed in Hoekzema et al. (2017). Scale bars = 10 mm.

Dickinsoniomorpha (Fig. 4) are defined as serially repetitive organisms with anterioposterior differentiation (Erwin *et al*., 2011 SOM), and include the genera *Dickinsonia*, *Yorgia*, *Windermeria* and *Andiva* (Erwin *et al*., 2011). However, there is divergence of opinion concerning the composition of this morphogroup, and alternative groupings have been proposed, some of which include taxa such as *Spriggina* (Dzik & Ivantsov, 1999; Grazhdankin, 2014). Dickinsoniomorph taxa are all restricted to broadly shallow‐marine settings ∼559–551 Ma (Waggoner, 2003; Boag *et al*., 2016).

Unlike the seemingly sessile rangeomorphs, dickinsoniomorphs, specifically *Dickinsonia* and *Yorgia waggoneri*, can be associated with impressions interpreted as trace fossils, suggesting a capacity for active locomotion (Ivantsov & Malakhovskaya, 2002; Gehling *et al*., 2005; Sperling & Vinther, 2010; although see McIlroy, Brasier, & Lang, 2009). Dickinsoniomorphs have been interpreted to exhibit evidence for internal anatomy, including gonads and diverticulae (e.g. Jenkins, 1992; Dzik, 2003), but such features have alternatively been interpreted as taphonomic artefacts (e.g. Brasier & Antcliffe, 2008). Constructional units in dickinsoniomorphs have been likened to metazoan segments (Wade, 1972), but more recent interpretations have argued that they may represent only external annulations (Sperling & Vinther, 2010), features invoked by some authors as the precursor‐state to a fully metameric bauplan (Chipman, 2010). Morphogenesis has been considered most commonly in *Dickinsonia costata* (e.g. Runnegar, 1982), a taxon that has been discussed in debates surrounding the evolution of bilaterality (Malakhov, 2004; Gold *et al*., 2015).

#### (a) Dickinsonia

*Dickinsonia costata* (Fig. 4D) has been described from shallow‐marine siliciclastic facies in South Australia and Russia. It exhibits an approximately oval outline, with distally expanding units emanating from a visible central midline. Units are continuous across the midline (Runnegar, 1982; Gold *et al*., 2015), imparting a bilateral symmetry. *D. costata* in Australia range from ∼6–250 mm in length (Reid *et al*., 2017), with size variants commonly considered to represent different ontogenetic stages (e.g.Evans *et al*., 2017 ; Hoekzema *et al*., 2017). Smaller specimens possess fewer units (as few as 12) than larger ones (which can have as many as 74; Sperling & Vinther, 2010). A triangular, undivided region seen in small specimens encompasses a proportionally smaller area of the body in increasingly larger specimens (the deltoidal region, e.g. Hoekzema *et al*., 2017), suggesting that in very early ontogenetic stages there may not have been any units at all (Ivantsov, 2007). The largest units are located close to the middle of the organism, not at either pole (Sperling & Vinther, 2010; Hoekzema *et al*., 2017). The position of the smallest units has often been used to infer the position of a growth zone (Runnegar, 1982; Ivantsov, 2007; Evans *et al*., 2017), which has been described as being in a ‘posterior’ position (Ivantsov, 2007) with units added terminally (Gold *et al*., 2015; Evans *et al*., 2017). Gold *et al*. (2015) follow Jacobs *et al*. (2005) in their definition of ‘terminal addition’, but figure a truly terminal generative zone (fig. 2 in Gold *et al*., 2015). Evans *et al*. (2017) do not define ‘terminal addition’, but reference Gold *et al*. (2015) and so we assume they also follow the definition of ‘terminal addition’ in Jacobs *et al*. (2005). However, recent work suggests that *Dickinsonia* instead added units at the opposing pole (Hoekzema *et al*., 2017). The latter authors characterise growth of units within populations of organisms interpreted to represent multiple ontogenetic stages, and present evidence for differentiation of new units from the margins of the undifferentiated region itself. In this scenario, which we support, the generative zone of *Dickinsonia* may be considered pre‐terminal (Fig. 4F). Further recent work has considered *Dickinsonia costata* to represent a paedomorphic variant of *Dickinsonia tenuis* (which possesses a greater unit count than *D. costata*; Zakrevskaya & Ivantsov, 2017).

These observations together suggest that *Dickinsonia* grew by the ‘insertion’ of new units, which then underwent subsequent inflation (see Runnegar, 1982; Fig. 4F). Larger specimens possess proportionally fewer units relative to their length, implying a reduction in the rate of unit addition (Evans *et al*., 2017; Hoekzema *et al*., 2017). However, there is variation in the number of units per specimen that is seemingly independent of (active?) contraction noted in many individuals (Evans *et al*., 2017). *Dickinsonia* has been conflictingly interpreted to show both a pre‐determined (Runnegar, 1982; Ivantsov, 2007) and an indeterminate (Retallack, 2007) mode of growth, but the apparent absence of size outliers belonging to *D. costata* appears to suggest that deterministic growth is more likely. The species *Dickinsonia rex*, however, could reach much greater sizes (∼43 cm; Jenkins, 1992), suggesting that a determinate pattern of growth cannot yet be assumed for all *Dickinsonia* species.

#### (b) Ontogenetic trends across dickinsoniomorphs

Unlike *Dickinsonia*, *Andiva ivantsovi* (Fig. 4A–C) is not bilaterally symmetrical, bearing a glide plane of symmetry along its axial midline. *Andiva* does possess an undivided region, but whereas in *Dickinsonia* this region appears to diminish in size as the organism grew, its proportions relative to the overall organism are seemingly maintained in *Andiva* (Fedonkin, 2002). *Andiva* differs from *Dickinsonia* in several other regards. For example, there is seemingly no clear relationship between specimen size and number of units. Like *Andiva*, *Yorgia waggoneri* (Fig. 4E) also appears to possess an undivided region at all known stages of growth (Dzik & Ivantsov, 1999; Ivantsov, 2007). The smallest *Yorgia* specimens possess 10–12 independent units, while larger specimens can have up to 70 (i.e. 35 ‘isomer pairs’; Ivantsov & Fedonkin, 2001) aligned along a glide plane of symmetry, *contra Dickinsonia*. If *Dickinsonia*, *Andiva* and *Yorgia* are closely related, it is fair to assume they would possess a similarly positioned generative zone. We find potential evidence that *Andiva* differentiated units from the opposite end to its undifferentiated area (i.e. its anti‐deltoidal pole, see Hoekzema *et al*., 2017), based on the recognition of an apparently partially differentiated unit (Fig. 4A–C). While this could be alternatively interpreted as two overlying units, if correct this observation suggests that in *Andiva*, differentiation occurred at a truly terminal generative zone, at the opposite end to the non‐differentiated region of the organism. Further work on a greater number of specimens is required, but it seems that the morphological differences previously outlined between *Dickinsonia* (bilaterally symmetrical with a proportionally variable deltoidal area) and *Andiva* (glide symmetry, and an undifferentiated crescentic region of fixed size relative to the body) may be corroborated by developmental differences, with growth progressing at different ends of the organisms with respect to their undifferentiated regions. Whether the undifferentiated deltoidal region of *Dickinsonia* and the crescentic region of *Andiva* are homologous remains to be determined. Our developmental comparisons do, however, raise the possibility that while *Dickinsonia* is arguably of the same morphological grade as other ‘dickinsoniomorph’ taxa, it may not ultimately belong to the same clade.

### (3) Erniettomorpha

**Figure 5:**
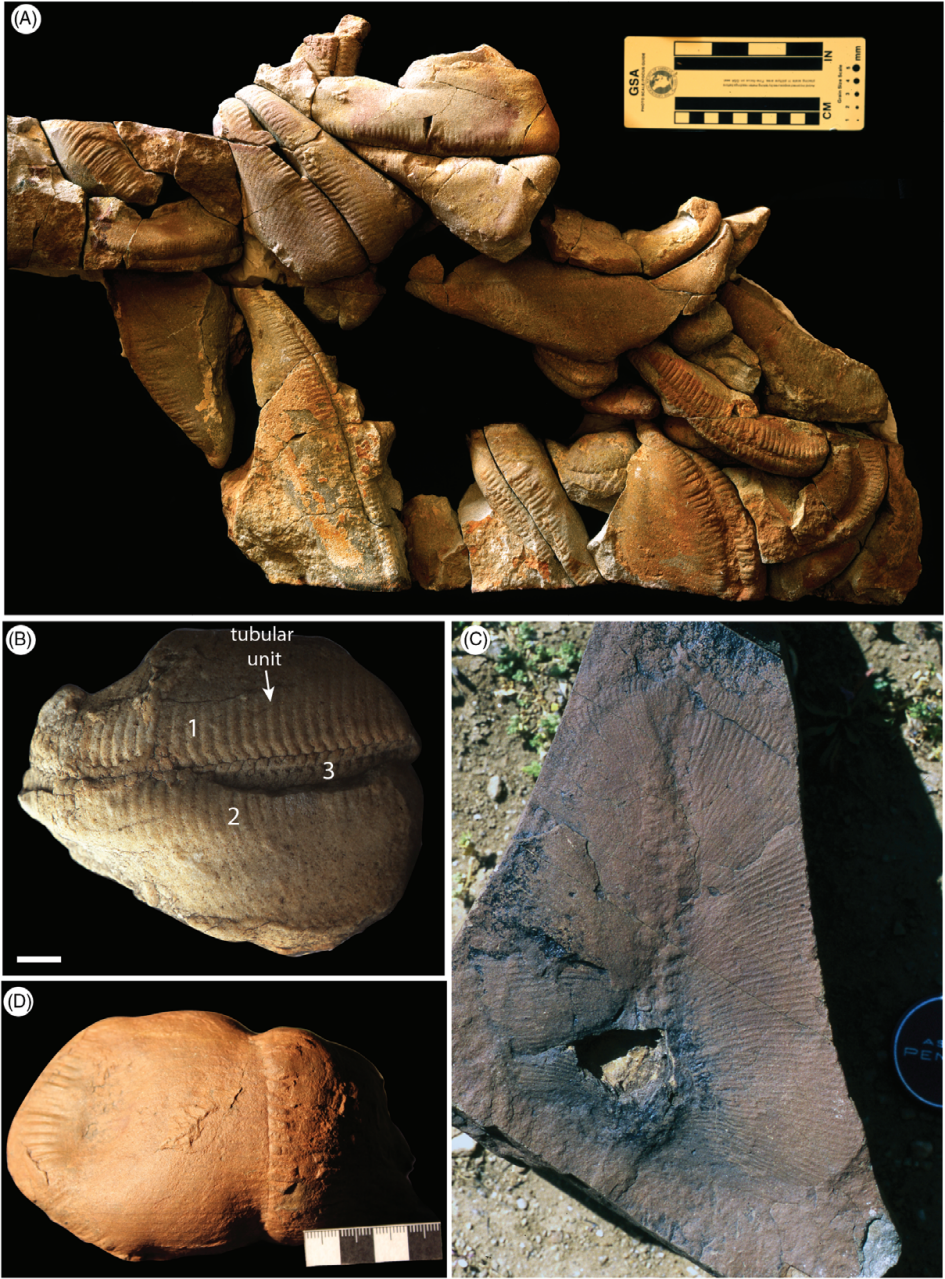
Ediacaran erniettomorph taxa. (A, B) Pteridinium simplex, Namibia. Numbers identifying the three identified vanes. (C) Swartpuntia germsii, Namibia. (D) Ernietta plateauensis, Namibia. Scale bars = 10 mm. Images courtesy of D. Grazhdankin (A and B from Grazhdankin & Seilacher, 2002), M.D. Brasier (C), and M. Laflamme (D).

Erniettomorphs (Fig. 5) are defined as serially repetitive organisms constructed entirely of tubular units arranged into fronds, ‘sac‐like’ or ‘canoe‐like’ benthic recliners, or flat‐lying mats (SOM of Erwin *et al*., 2011); this definition clearly encompasses a broad range of morphologies. Erniettomorphs are prominent constituents of the latest Ediacaran macrofossil assemblages of Namibia (∼550–541 Ma) (Darroch *et al*., 2015; Boag *et al*., 2016), and Nevada (Smith *et al*., 2017), yet their biology is little understood. Only two taxa, *Ernietta plateauensis* (a sac‐like form) and *Pteridinium simplex* (a canoe‐like form), have undergone recent detailed study (Elliott *et al*., 2011, 2016; Ivantsov *et al*., 2016). *Pteridinium simplex* is the most widely studied erniettomorph from an ontogenetic perspective, but whether its growth strategy is broadly applicable to all erniettomorphs is debatable given the morphological disparity of this group.

#### (a) Pteridinium simplex

*Pteridinium simplex* (Fig. 5A, B) appears to have been constructed of three vanes of tubular units (Fig. 5B) that meet in an alternating fashion at a central ‘seam’, imparting a glide plane of symmetry (Grazhdankin & Seilacher, 2002; Meyer *et al*., 2014). Complete specimens range from 6.0 cm in length (along the central seam, displaying 26 units) to 19.2 cm (with 55 units) (Grazhdankin & Seilacher, 2002). The number and length (long axis) of individual units appears to correlate linearly with the organism's total length, but the height of the organism (the distance between the central seam and the termination of the long axis of the units) does not follow a similar relationship (Grazhdankin & Seilacher, 2002). The relationship between unit length and overall length reveals two distinct morphological groupings of *Pteridinium*; one showing a positive correlation between the two variables, and one showing no correlation (Grazhdankin & Seilacher, 2002). This ontogenetic variation may imply the presence of two distinct *Pteridinium* species, or may alternatively hint at ecophenotypic variation within the taxon (the study of which amongst the Ediacaran macrobiota remains in its infancy: Kenchington & Wilby, 2017; Hoyal Cuthill & Conway Morris, 2017).

Specimens of *Pteridinium* can taper at one or both ends, with the tapering tip previously inferred to be the growth tip (Grazhdankin & Seilacher, 2002; Laflamme, Xiao, & Kowalewski, 2009). *Pteridinium* has thus been variously considered as both unipolar (Grazhdankin & Seilacher, 2002) and bipolar (Laflamme *et al*., 2009), although the lack of a tapering tip in some specimens may reflect a taphonomic bias (Seilacher, 1989). The distal‐most unit can be positioned on either side of the central seam, suggesting that *Pteridinium* added units sequentially across its different vanes (Tojo *et al*., 2007; although see Laflamme *et al*., 2009). *Pteridinium* has previously been considered to grow mainly by the ‘insertion’ of new units over time (Laflamme *et al*., 2009), but it appears that one morph also grew by the observable inflation of pre‐existing units (Grazhdankin & Seilacher, 2002). Specimens that are ∼6 cm long have been inferred to be immature (Grazhdankin & Seilacher, 2002), but there are no documented specimens of comparable size to those of the smallest rangeomorphs and dickinsoniomorphs (i.e. 10 mm or less).

#### (b) Ontogenetic trends across the erniettomorphs

The only other erniettomorph for which there is sufficient data to deduce ontogenetic information is *Ernietta plateauensis* (Fig. 5C). Unlike *Pteridinium*, the number of units remains relatively constant (23–28 on either side of the organism) across specimens of 35–55 mm in basal width (known size range 30–80 mm in width; Bouougri *et al*., 2011). This suggests that growth took place primarily by the inflation of units, rather than by their continued insertion, at least in larger specimens (Ivantsov *et al*., 2016). However, there has been considerable debate as to what constitutes a ‘juvenile’ *Ernietta* (Hahn & Pflug, 1985; Runnegar, 1992; Schopf & Klein, 1992; Elliott *et al*., 2016), and so we refrain from presenting an ontogenetic analysis of this taxon. Other erniettomorph taxa, such as *Swartpuntia* (Fig. 5D) (Narbonne, Saylor, & Grotzinger, 1997), have received relatively little attention in terms of their morphogenesis. Before the morphogenesis of erniettomorphs can be reliably assessed, a re‐evaluation of what constitutes membership of this group is required. Consequently, it is currently not possible to compare ontogenetic processes between the erniettomorphs, and thus evaluate the utility of this morphogroup.

## IV. DEVELOPMENTAL COMPARISONS AND PHYLOGENETIC INFERENCE

### (1) Extant taxa

**Figure 6:**
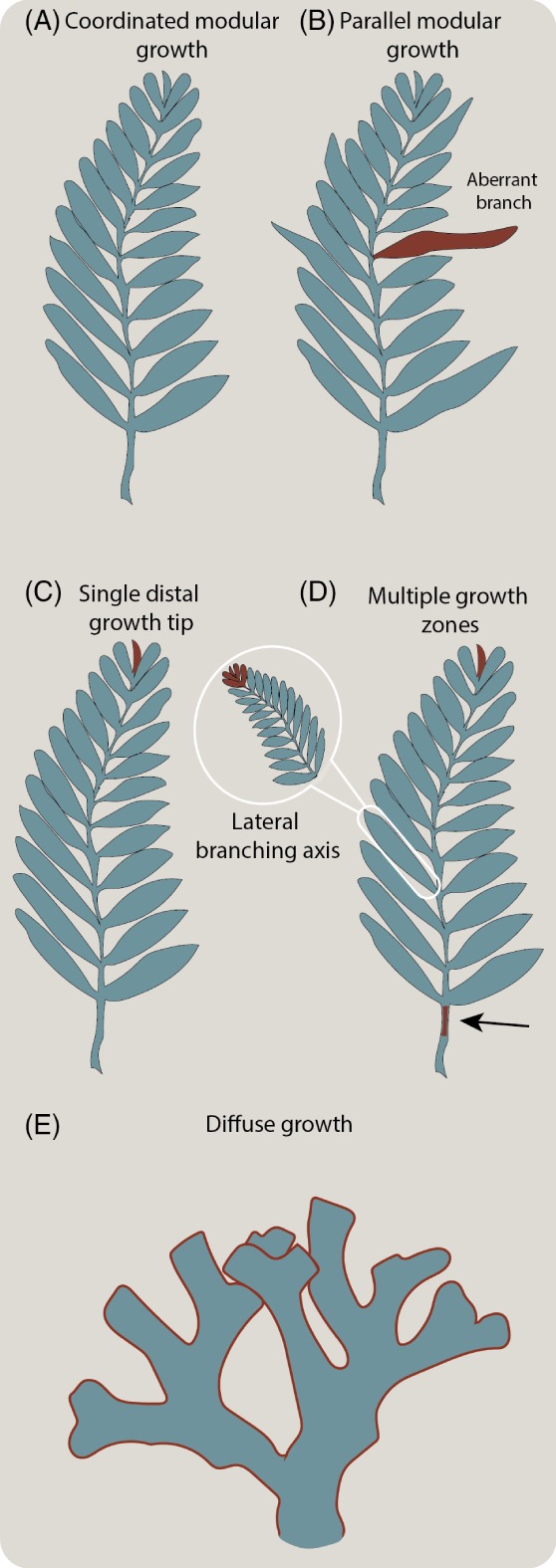
Schematic diagram showing the forms of growth observed in extant clades with serial repetition of component units; red indicates the style/feature of growth discussed. (A) Coordinated modular growth, seen in certain metazoan groups. (B) Parallel modular growth, common in plants and red, green and brown algae, with an aberrant branch highlighted in red. (C, D) Positioning of different central (additional growth zone highlighted with black arrow) and lateral growth zones/tips in extant serially repetitive groups. Single apical axes are seen in green and red algal groups, whereas multiple axes are seen in various metazoan and brown‐algal groups. (E) Diffuse growth, as seen in colonial bilaterian groups characterised by colony‐wide tip growth.

Among the eukaryotes, serial repetitive growth is known in the chlorophyte, streptophyte, rhodophyte, and phaeophyte algae, land plants, fungi, and members of the Metazoa (Gold *et al*., 2015). However, the processes by which these groups attain their essentially similar morphologies are very different. Plants and algae (red, green and brown) possess apical meristems, with the repeated re‐specification of lateral organs along their length (Kuhlemeier, 2007). Each lateral organ displays developmental independence and, as such, these groups are classified as modular, displaying parallel modular growth, which results in an indeterminate morphology (Kaandorp, 2012; Fig. 6A–B). Brown algae, unlike plants and other algal groups that possess only one axial growth zone (Fig. 6C), can possess multiple axial growth zones located more basally (intercalary meristems: Charrier, le Bail, & de Reviers, 2012; Fig. 6D). Brown algal intercalary meristems have been interpreted as derived, whereas the apical meristem is considered plesiomorphic (Charrier *et al*., 2012).

Fungi are also modular and grow from the tips of hyphae (Brand & Gow, 2009), but unlike the plants and the algae they lack a truly organismal body axis. Hyphae come together to form a fruiting body, rather than modules developing from a central structure as in plants. Moreover, fungi do not exhibit differentiation of new units over time. The fruiting body emerges following the formation of a ‘hyphal knot’ by multiply branched hyphae, and subsequently differentiates into the constituent parts (e.g. in the button mushroom Agaricus bisporus; Umar & Van Griensven, 1997).

While not serially repetitive, since a lichen affinity has been advanced for members of the Vendobionta (Retallack, 1994), their morphogenesis must be considered. Lichens are known to exhibit an indeterminate form, and so display parallel modular growth (e.g. fig. 1 in Suetina & Glotov, 2010).

Serial repetition is achieved in plants and algae by the presence of a totipotent meristem (a zone of cell proliferation that gives rise to the organs and tissues of a plant), but in colonial animals it can be achieved in a number of different ways. Within Cnidaria, coloniality is widespread in the anthozoans and the hydrozoans, and with two main mechanisms of colonial growth at play. Monopodial growth is much like the meristematic growth seen in plants, whereby growth proceeds primarily from an (sub)apical growth tip; in athectate hydrozoans, lateral branches are specified successively and these then display monopodial growth themselves. In thectate hydroids, this same pattern of monopodial growth cannot occur due to the presence of the theca. In these forms, the apical stem tip acts in a fashion similar to a meristem, specifying new lateral shoots on both sides of the organism simultaneously (Berking, 2006). Sympodial growth involves the cessation of growth at the apical growth tip, and the re‐specification of the ‘apex’ as outgrowths from successive lateral growth tips (Berking, 2006). Both monopodial and sympodial growth can occur either separately or concurrently. Some colonial anthozoans do not exhibit classical monopodial growth, with new branches emerging from a basal and pre‐terminal growth zone in Pennatulacea (Antcliffe & Brasier, 2007). Colonial cnidarians are also known to show colony polymorphism (discontinuous variation in zooid morphology within colonies: Hyman, 1940a; Boardman, Cheetham, & Oliver, 1973). In such cnidarians, repeated units tend to appear in sets, or whorls (Gold et al., 2015).

Extant members of Porifera do not show a serially repetitive body plan in the same way as certain cnidarians, and do not display the same level of colonial integration (i.e. the division of labour). However, certain sponges (e.g. the demosponge Callyspongia vaginalis) are constructed of serially repeated units. Recent work has elucidated a broad repertoire of developmental regulatory genes in the Porifera, hinting at ancestral complexity in the early sponges (Leininger et al., 2014). While Placozoa has been considered sister to Bilateria (Collins, 1998), recent work suggests that the cnidarians are sister to Bilateria (e.g. Cannon et al., 2016). No‐one has yet reconstructed the ancestral states of Placozoa (or Ctenophora for that matter), and the presumably simplified morphology of extant placozoans, and the derived nature of extant ctenophores, means we should not exclude either group from the Ediacaran debate.

Many colonial bilaterians (belonging to Rouphozoa and Gnathifera; Laumer et al., 2015) tend to show, in the broadest sense, a more diffuse form of colonial growth (Fig. 6E). In bryozoans, which can possess frondose or arborescent forms, new zooids emerge by budding, with the pattern of budding being almost species specific and determining the form of the colony (Hyman, 1940b). The entoprocts, once considered to be members of Bryozoa, are largely colonial in form. Rather than taking an arborescent form, entoprocts often grow through laterally spreading stolons, with vertically projecting zooids emerging at intervals. Meanwhile the rotifers display an aggregative form of colonialism, whereby juveniles become tangled up and eventually adhere to each other by production of an adhesive string from a foot gland (Surface, 1906).

The serially repetitive structures observed in members of the segmented unitary Bilateria – the arthropods, annelids and chordates – develop largely through the process of posterior growth via the specification of units in parallel with the elongation of the anterior–posterior axis (Jacobs et al., 2005). Whereas in many serially repetitive organisms there is a disjunct between the growth of individual units and the growth of the main body axis, the two are concurrent in the segmented Bilateria. The specification of units is sequential in most of these bilaterians, but there are exceptions, such as the long‐germ‐band insects (e.g. Drosophila melanogaster), which specify the entire anterior–posterior axis simultaneously (Liu & Kaufman, 2005). The patterns imparted by different forms of segmentation can manifest in different ways. Organisms can be homonomously segmented, whereby segments are largely identical, or groups of segments performing similar tasks may group together into functional units known as tagmata.

### (2) Implications for the Ediacaran macrobiota

Proposed members of the rangeomorphs, dickinsoniomorphs and erniettomorphs have all been described as growing by either the differentiation of new units, the inflation of pre‐existing units (at known ontogenetic stages), or a combination of the two (Table 1). Description of growth by the differentiation of new units and/or their subsequent expansion alone is, however, uninformative for constraining phylogenetic affinity, since this method of formulating new units is universal among multicellular eukaryotic groups (Bonner, 1952). The absence of data on the very earliest growth stages (of a few millimetres or less) in Ediacaran taxa also hampers efforts to determine the point at which differentiation occurred in the life cycle in some taxa.

The position of the generative zone is potentially a more useful developmental character, but identification of this trait in rangeomorphs, dickinsoniomorphs, and erniettomorphs remains difficult since the assumption that the position of the smallest units correlates with the position of the generative zone has recently been questioned (Hoekzema et al., 2017). In the following discussion, we assume that previously ascribed generative zones as discussed in the above sections are correct, but note that such assumptions remain unproven.

Rangeomorphs exhibit a non‐deviant form (i.e. aberrant‐length branches have not been observed in thousands of studied specimens). It is, therefore, highly likely that rangeomorphs do not exhibit the parallel modular growth characteristic of non‐metazoan serially repetitive groups. Their shape is seemingly constrained at both the organismal level, and at the level of individual branches (including subsidiary branches; Gehling & Narbonne, 2007), across the known ontogenetic series.

**Figure 7:**
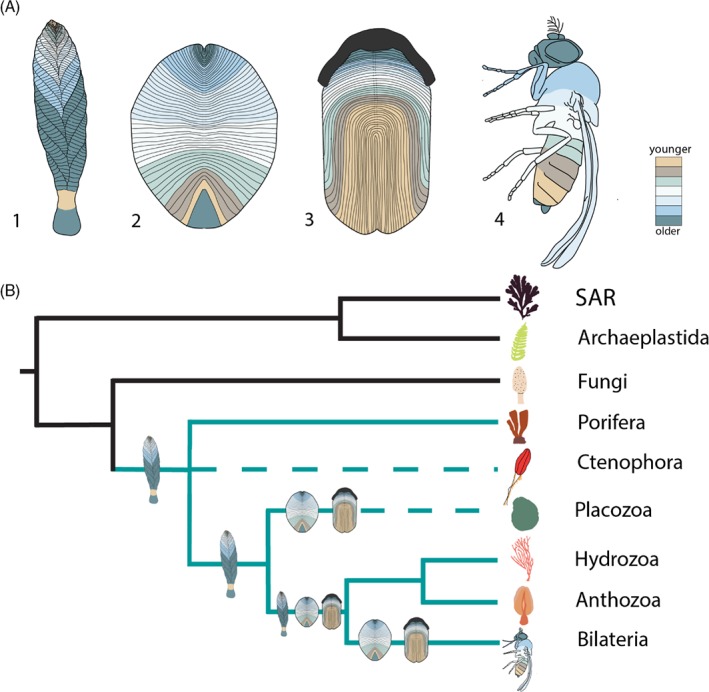
(A) Interpretive growth models of: 1, Charnia masoni; 2, Dickinsonia costata; 3, Andiva ivantsovi; 4, an extant bilaterian comparator. (B) A simplified eukaryote phylogeny including only groups with serially repetitive body plans to which the Ediacaran morphogroups have been compared. SAR = Stramenopiles, Alveolates and Rhizaria. The suggested phylogenetic positions of Charnia, Dickinsonia and Andiva are presented as discussed in the text (we include Andiva as possibly being resolved within the Bilateria because although our morphological data may suggest a truly terminal generative zone, this is based on one specimen and additional data are required to confirm or refute this). Green represents metazoan lineages. Dashed lines indicate the possible position of a group (owing to uncertainty surrounding the phylogeny of the basal Metazoa; e.g. Dunn et al., 2014).

Unlike Fungi, rangeomorphs exhibit the differentiation of new units. The presence of a basal growth zone (in the stem and potentially in some of the lowermost primary branches), as well as an apical one, at least in Charnia, would ally them to Eumetazoa (but of course our understanding of plesiomorphic states in early diverging metazoans is wanting). The presence of discrete (as opposed to diffuse) growth tips would argue against affinities with most members of Rouphozoa and Gnathifera, but the likely presence of multiple axial growth zones (in Charnia) and potential secondary growth tips (in Bradgatia), is reconcilable with known variation in members of the colonial cnidarians. Based on current data, we cannot rule out a stem‐metazoan affinity for rangeomorphs (if Porifera are the sister lineage to all other metazoans; Pisani et al., 2015), or, indeed, a stem‐poriferan affinity, but the general paucity, as opposed to conflict, of data prevents further assessment (Fig. 7). We do not consider a ctenophore affinity likely since both extant ctenophores and organisms considered to be stem‐ctenophores, including the Ediacaran Eoandromeda, are considered to be motile (Tang et al., 2011).

Dickinsoniomorphs as currently defined also lack evidence of parallel modularity, and show the differentiation of new units across ontogeny, precluding algal and fungal phylogenetic affinities. When combined with trace fossil evidence for motility, and anatomical evidence (Sperling & Vinther, 2010), this developmental constraint likely requires that they are metazoan. The data of Hoekzema *et al*. (2017) suggest that *Dickinsonia* may have possessed a pre‐terminal growth zone along with concurrent inflative growth in lateral units and the main growth axis, which can be reconciled with the basal and pre‐terminal generative zone of extant segmented bilaterians (Fig. 7A). There are, of course, exceptions to this rule, such as Onychophora (which grow from a true terminus; Anderson, 1973), or Nematoida (where a secondary loss of serially repetitive units makes confirmation of a pre‐terminal growth zone difficult), but these conditions have been considered to be derived from an ancestral pattern of pre‐terminal addition (Jacobs *et al*., 2005). The monopodial serially repetitive cnidarians also show a pre‐terminal mode of extension rather than a true terminal growth zone, so a pre‐terminal generative zone for *Dickinsonia* remains compatible with such organisms. However, organisms of cnidarian grade may also exhibit truly terminal differentiation (e.g. monopodially growing athectate hydrozoans; Berking, 2006). A placozoan affinity for *Dickinsonia* (Sperling & Vinther, 2010) is difficult to evaluate on developmental grounds given the low diversity and disparity of extant placozoans, and remains a viable possibility (Fig. 7). The potential for a truly terminal growth zone in *Andiva* (Fig. 7) could, however, suggest that a non‐bilaterian affinity is possible for at least some dickinsoniomorph taxa.

Currently, the erniettomorphs are too poorly understood to infer their phylogenetic position from developmental data. Members of Erniettomorpha have been considered to show morphological similarities to members of the annulated *Dickinsonia*‐like taxa (e.g. Budd & Jensen, 2017), but whether this evidences a phylogenetic relationship is unclear. The relative consistency of overall form in erniettomorphs suggests that they do not exhibit parallel modular growth and, thus, they are unlikely to be plants or algae. Continuous differentiation of new units in *Pteridinium* seemingly rules out a fungal affinity. There are no current data to exclude *Pteridinium* from Metazoa, but there is similarly no additional evidence to support a metazoan affinity. Given our poor knowledge of erniettomorphs, we cannot currently extrapolate from *Pteridinium* to other organisms. Indeed, this review has highlighted significant gaps in knowledge of development in multiple Ediacaran taxa, as well as taxonomic issues that require resolution before morphogenesis can be meaningfully addressed in other morphogroups.

## V. IMPLICATIONS FOR DEVELOPMENTAL EVOLUTION

Developmental evidence supports a metazoan affinity for rangeomorphs (Fig. 7B). Their multiple axial growth zones, as well as their asymmetric glide plane of symmetry, apparent in all known life stages, argue against most bilaterian affiliations, but we note that forms of glide symmetry are known in bilaterian taxa including echinoids (e.g. between plates in the interambulacral zone) and graptolites (e.g. *Eoglyptograptus*). There are also rare reports of bilateral symmetry at higher branching orders in some rangeomorphs (figs 3D, 4A, 5C in Flude & Narbonne, 2008), potentially revealing complexity in the axial patterning of these organisms, and illustrating that symmetry may not represent a reliable phylogenetic indicator for Ediacaran taxa.

The rangeomorphs appear to have one main body axis and one lateral branching axis, an arrangement very similar to various cnidarian organisms (Watanabe *et al*., 2014), with which they also share developmental similarities, i.e. a conserved form and potential positioning of the generative zone. The possibility that rangeomorphs possessed a third body axis (akin to the dorso‐ventral axis), cannot yet be excluded, but seems unlikely given evidence to suggest that some rangeomorphs were identical on both ‘sides’ (e.g. fig. 3 in Seilacher, 1992; fig. 5.2 in Wilby *et al*., 2015; although see Gehling & Narbonne, 2007, for a discussion of taphonomic reasons for why a third vane may not be preserved in *Fractofusus*). Sponges are conventionally interpreted to possess just one principal body axis, but a reduction in the number of body axes may be a consequence of simplification (e.g. Ferrier, 2015). Therefore, resolution of the rangeomorphs as falling within the metazoan stem or, indeed, total‐group Porifera, cannot be excluded.

The rangeomorphs do not show either true radial symmetry or bilateral symmetry, but the possibility that rangeomorphs like *Charnia* displayed biradial symmetry could prove informative. If the rangeomorphs belong to the eumetazoan stem, their possible possession of biradial symmetry could support the notion that biradiality was a precursor to bilateral symmetry in metazoans (Martindale & Henry, 1998). This is particularly pertinent given that the rangeomorphs may themselves have possessed bilateral symmetry at smaller branch orders (Flude & Narbonne, 2008). Alternatively, tentative biradial symmetry could support the idea that early metazoans experimented with variants of radial symmetry independent of phylogeny (see also the putative stem‐ctenophore *Eoandromeda* which exhibits octoradial symmetry, the triradial form *Tribachidium*, tetraradial *Conomedusites*, and pentaradial *Arkarua*; Xiao & Laflamme, 2009).

*Dickinsonia*, like rangeomorphs, appears to possess one major body axis and one lateral axis, with insufficient evidence to determine differentiation across a third axis [although see Evans *et al*. (2017) for discussion of *Dickinsonia* ‘height’]. We resolve *Dickinsonia* as a member of total‐group Metazoa (Fig. 7B), likely within the Placozoa plus Eumetazoa total group, on the basis of the developmental evidence presented above, combined with the apparent capacity for active locomotion (see Hoekzema *et al*., 2017).

Consideration of *Eoandromeda octobrachiata* as a stem‐ctenophore (Tang *et al*., 2011) has resulted in attempts to find homology between the body axes of radial and non‐radial Ediacaran taxa. The asymmetric head region of *Yorgia* has been speculatively likened to two of the three branch‐like structures that make up *Tribrachidium* (Budd & Jensen, 2017), implying axial homology between the dorso‐ventral axis of *Tribachidium* and the ‘anteroposterior’ axis of dickinsoniomorphs. In the absence of an asymmetric undivided region in some dickinsoniomorphs, and even in some *Dickinsonia* specimens, we do not consider that there are sufficient grounds to consider these axes to be homologous.

If members of the Dickinsoniomorpha can be resolved with bilaterians, they may prove informative on the appearance of bilaterian characters. In the evolution of metamerism, a determinate form (i.e. a pre‐determined number of units) likely appeared late; well after the initial appearance of true metamerism (Vroomans, Hogeweg, & Tusscher, 2016). In *Dickinsonia*, organisms of different sizes display variable numbers of units, such that the number of units does not appear pre‐determined (Evans *et al*., 2017; Hoekzema *et al*., 2017). Therefore, if *Dickinsonia* was truly metameric (and future work is required to establish this), the fossil data would appear to concur with these prior theoretical predictions. Interestingly, the positions of putative internal anatomical structures preserved within *Dickinsonia* (e.g. Dzik & Ivantsov, 2002; Zhang & Reitner, 2006) do not correlate with the positions of the visible units considered to be on the exterior of the organism. As such, if these structures represent true biological features, and these organisms were truly segmented, they must have been heteronomously so (i.e. where segments are non‐identical), possessing tagmata. While it is likely that the three main segmented bilaterian groups all developed segmentation independently of each other, it appears that the homonomous state is plesiomorphic to the arthropods and annelids (being present in the stem‐lineages of these clades if we discount highly derived tagma in the head regions; e.g. Parry, Vinther, & Edgecombe, 2015; Ortega‐Hernández, Janssen, & Budd, 2016), whereas heteronomous segmentation appears plesiomorphic to the vertebrates [for example, in the vertebral column (Jacobs *et al*., 2005)]. We therefore find that dickinsoniomorphs do not sit comfortably in the stem lineages of annelids or arthropods on account of their seemingly heteronomous state. However, the absence of any chordate diagnostic characters means they cannot be reconciled with chordates either. Therefore, if members of Dickinsoniomorpha are resolved as being segmented, in this scenario we consider it most likely that they represent a bilaterian group that independently adopted a segmented form.

Another consideration is that some dickinsoniomorphs (perhaps most notably *Yorgia*) exhibit glide symmetry, not bilateral symmetry, meaning that under the scenario in which the dickinsoniomorphs do represent a coherent clade, any ‘segments’ would be discontinuous across the midline. Two possibilities then arise: *Yorgia* is not segmented, but does possess external annulations that may or may not be a precursor state to true segmentation; or conversely, *Yorgia* does display a form of derived segmentation similar to that seen in long‐germ‐band insects today, where the ‘segments’ are not the fundamental unit. In these cases, parasegments cross segment boundaries (Martinez‐Arias & Lawrence, 1985), and pattern the embryo of certain insects (e.g. *Drosophila*).

The resolution of these organisms as falling within Metazoa does not in itself help us to resolve between their potential body axes. It is broadly true that sponges have one main body axis, diploblasts have two and triploblasts have three, and that these main axes are patterned by the same pathways and gradients, and so may be homologous (e.g. Leininger *et al*., 2014). Wingless‐related integration site (Wnt) patterning across both the oral–aboral and anterior–posterior axes (e.g. Holstein, 2012) may suggest that the primary axis across Eumetazoa is homologous, and similar Wnt patterning across the primary body axis of sponges suggests that the primary body axis across all Metazoa may be homologous (Leininger *et al*., 2014). Similarly, bone morphogenetic protein (BMP) signalling across the directive and dorso‐ventral axes (Matus *et al*., 2006; Genikhovich *et al*., 2015) may or may not suggest homology across Eumetazoa. However, many animal groups show major shifts in axial patterning, and so using morphology alone can lead to difficulty in identifying even analogous axes (e.g. the secondary acquisition of a pentameral body plan in starfish and sea urchins confounds identification of the anterior–posterior axis). Cnidarians, as a group, are almost typified by a number of excursions into radial symmetry (perhaps from a bilateral ancestor; Dzik, Baliński, & Sun, 2017), making the directive axis hard to identify from morphology alone. There are also examples of organisation along the dorso‐ventral axis being inverted between arthropods and vertebrates [i.e. the reversal of positioning of the nerve cord (e.g. Denes *et al*., 2007)]. Many Ediacaran macro‐organisms inferred to represent ancient animals are themselves characterised by excursions into forms of radial symmetry, potentially independent of phylogeny, making points of homology difficult to ascertain. If axis homology can be proven by resolution of phylogenetic placement, these fossils could be interpreted to represent a primitive diversity of body plans, perhaps suggesting that successive disruptions and alterations to the planes of these body axes may be plesiomorphic. However, these data also warn of the problems of inferring homology across the body axes of diploblasts and triploblasts; if *Dickinsonia* is resolved as being a placozoan, or cnidarian, then definition of its main body axis as anterior–posterior (e.g. SOM of Erwin *et al*., 2011) is inappropriate. Until axis homology can be identified, it seems prudent to use phylogenetically neutral terms to describe body axes.

## VI. CONCLUSIONS

(1) There is significant potential to improve our knowledge of development in Ediacaran macro‐organisms, but the synthesis of existing data allows us to refute several previously proposed phylogenetic affinities for key Ediacaran taxa. Analysis of development in rangeomorphs and dickinsoniomorphs reveals congruence with aspects of metazoan development.

(2) We conclude that developmental data alone allow us to identify *Dickinsonia*, *Andiva*, *Yorgia* and the rangeomorphs as early metazoans.

(3) Morphogenesis offers promise for disentangling Ediacaran phylogenetic relationships and the evolution of development. Although the study of ontogeny is the study of change over time, by adopting a largely morphological approach when considering Ediacaran organisms, the ‘change’ has been largely overlooked. Future study of populations of organisms will allow better quantification of this change, as well as the production of growth models, both of which will ultimately increase the precision of phylogenetic resolution of Ediacaran organisms.

(4) The recognition of some the most enigmatic members of Ediacaran fossil assemblages as probable metazoans offers support to recent suggestions of considerable developmental complexity in early‐branching metazoans (e.g. Ferrier, 2015), and lends credence to the idea that the early metazoan tree cannot be rationalised in terms of gradually increasing complexity, but may have followed a much more cryptic path.

## VII. ACKNOWLEDGEMENTS

F.S.D., P.C.J.D and A.G.L. are funded by the Natural Environment Research Council [NE/L002434/1, NE/P013678/1, NE/L011409/2]; P.C.J.D is also funded by BBSRC [BB/N000919/1], The Royal Society, and a Royal Society Wolfson Research Merit Award. The authors thank Andrey Ivantsov for permission to figure new material, held at the Palaeontological Institute (Moscow), and Guy Narbonne, Graham Budd and anonymous reviewers for insightful comments. The authors confirm that they have no conflicts of interest.
